# Anthropometric Analysis of Cuboid Bones in a South Indian Population

**DOI:** 10.7759/cureus.51622

**Published:** 2024-01-03

**Authors:** Sulochana Sakthivel, Yuvaraj Maria Francis, Sankara N G, Sarala D K. V, Nithya Dhakshnamoorthy

**Affiliations:** 1 Anatomy, Jawaharlal Institute of Postgraduate Medical Education and Research, Puducherry, IND; 2 Anatomy, Saveetha Institute of Technical and Medical Sciences, Chennai, IND; 3 Anatomy, Saveetha Medical College and Hospital, Chennai, IND; 4 Anatomy, Employees' State Insurance Company Medical College and Hospital, Chennai, IND

**Keywords:** os peroneum, navicular articulating facet, calcaneal articular facet, biometry, cuboid bone

## Abstract

Purpose

Cuboid bone and its fibromuscular supports maintain the lateral longitudinal arch in weight transmission during different gait cycle phases. Morphometry of the cuboid bone is essential for designing a cuboid prosthesis for foot reconstruction and establishing an individual’s biological profile. The present study aims to assess the morphology and morphometry of the cuboid bone.

Materials and methods

The study used 103 cuboid bones (right 50, left 53) of unknown sex. Different shapes of cuboid articular facets were observed, and the morphometric parameters such as length, breadth, and height of cuboid, and the dimensions of articular facets in cuboid (calcaneal facet, fourth and fifth metatarsal facets, ecto-cuneiform facet, navicular facet, and facet for os peroneum) were analyzed.

Results

The mean length, breadth, and height of the cuboid bone were 33.69 ± 2.61 mm, 25.43 ± 2.87 mm, and 23.03 ± 2.43 mm, respectively. The mean transverse and vertical diameters were 23.22 ± 2.4 mm and 15.97 ± 1.85 mm, respectively. Facet for os peroneum was observed in 74.76% and for navicular bone in 26.2%. The mean transverse and vertical diameters were 7.16 ± 2.08 and 6.78 ± 1.78 mm, respectively. The depth of the peroneal groove was 4.30 ± 1.11 mm.

Conclusion

The morphometric data from the present study could assist in preoperative planning and designing of prostheses for foot reconstruction, and in establishing the biological profile of an individual, which can help the anthropologists in identifying the unknown remains.

## Introduction

Morphology of unknown human skeletal remains can be a superior analytical tool for biological profiling with less ambiguity, particularly in fragmentary skeletal remains, when provided with reference to the collection of bone data sets with known racial features and sexual dimorphism [1]. The tarsal bones have the compelling property of a lower rate of decomposition. The identification and sex determination of human skeletal remains can be done more easily in tarsal bones than in other bones [2].

The cuboid bone is the third-largest tarsal bone inserted between the calcaneus proximally and the fourth and fifth metatarsals distally. This peculiar bone articulates with five of the seven tarsal bones, forming the mid-tarsal joints with integrity maintained by ligaments. The cuboid acts as a keystone for the stability of the lateral longitudinal arch and lateral column of the foot [3]. Cuboid is always associated with adjacent tarsal bone pathology, resulting in chronic foot pain syndromes, including cuboid syndrome, os peroneum syndrome, peroneal tendonitis, and stress fractures in cuboid and metatarsal bones [4]. 

The morphology and morphometric analysis of cuboid bone is crucial for designing the prosthesis for foot and ankle reconstructions and in finite element modeling (FE) or statistical shape modeling (SSM) [5, 6]. Knowledge about cuboid morphometry can assist surgeons in understanding the basics behind lateral foot syndrome, which includes chronic pain, trauma, fracture dislocation, and joint instability associated with cuboid bone, and in cuboid osteotomy for lateral column lengthening in flatfoot deformity [7]. It also provides knowledge about the evolution of human bipedalism and the kinematics of the cuboid bone with adjacent tarsal and metatarsal bones, which can help during prosthesis design. This study aims to evaluate the morphology of the cuboid bone, including the morphometry of its articular facets. This will aid in the design of a cuboid bone prosthesis for reconstructive foot and ankle surgeries.

## Materials and methods

This descriptive study was done after the approval of the Institutional Review Board of Saveetha Medical College and Hospital (SMC/IEC/2022/05/125), utilizing 103 cuboid bones (right 50, left 53) that were available for research in the Department of Anatomy during the study period of January 2022 to August 2023. These bones were collected from cadavers of South Indian origin. The age and gender of the bones were unknown.

The bones included in this study were procured from the buried cadaver after one year. The bones were treated using the standard procedure to remove the fibrocartilagenous remnants and wood varnished to maintain the shelf life. The bones with intact morphology were included in this study, and bones with structural abnormalities, macroscopic lesions, or scars because of decay and trauma were excluded from this study.

**Figure 1:**
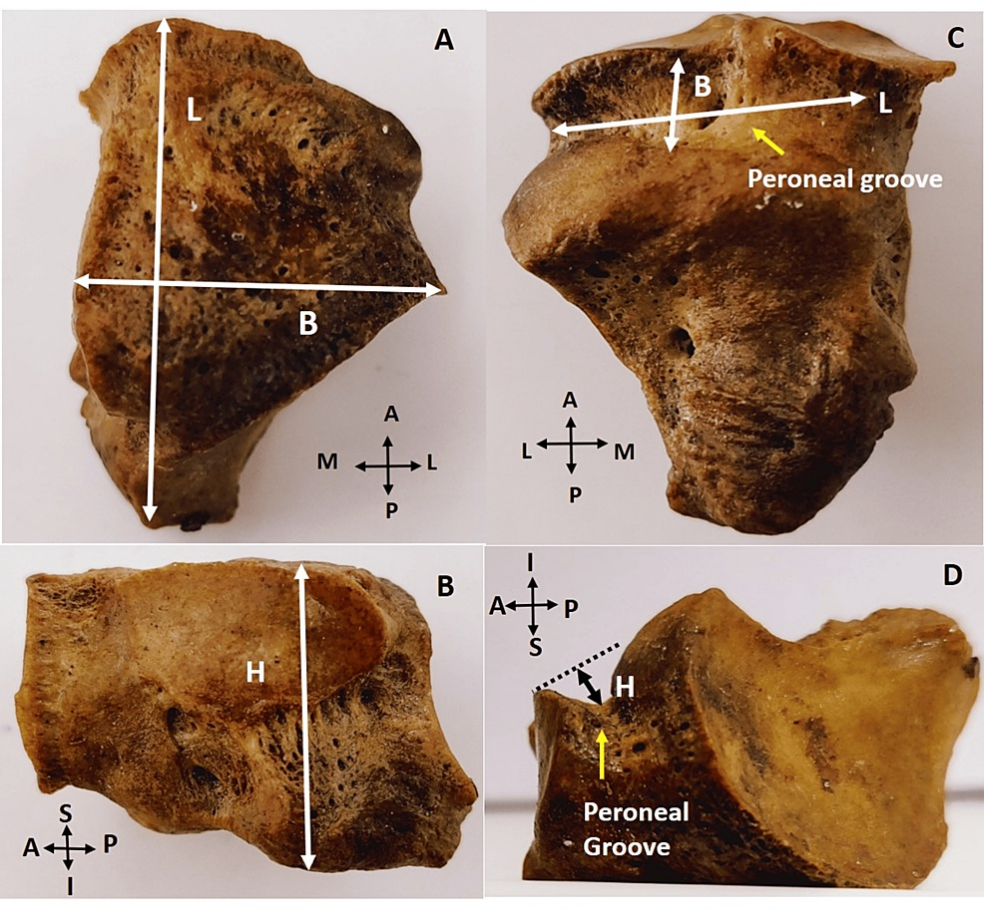
Dimensions of right cuboid bone and peroneal groove A) superior view: length and breadth of cuboid bone; B) medial view: height of cuboid bone; C) inferior view: length and breadth of peroneal groove; D) lateral view: height of peroneal groove L - length; B - breadth; H - height

**Figure 2:**
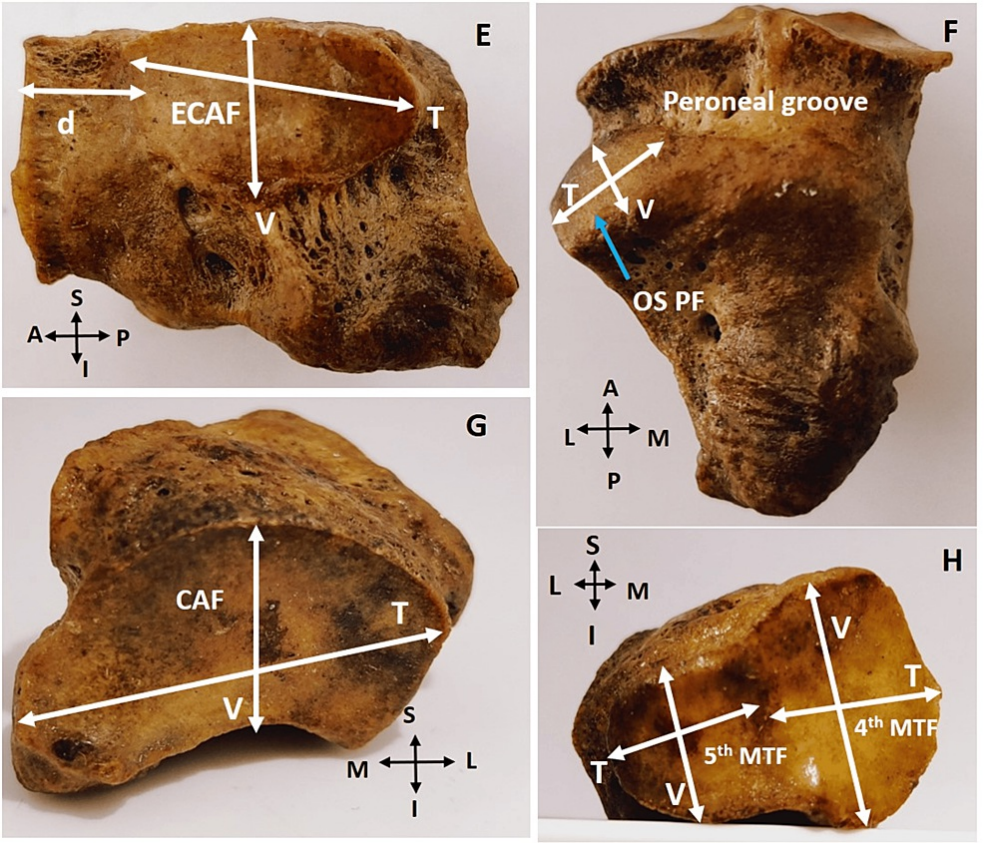
Dimensions of articular facets of right cuboid bone and peroneal groove E) medial view: dimensions of ecto-cuneiform articular facet (ECAF) and its distance (d) from metatarsal facet; F) inferior view: dimensions of facet for os peroneum (OS PF); G) posterior view: dimensions of calcaneal articular facet (CAF); H) anterior view: dimensions of fourth and fifth metatarsal facets (fourth MTF and fifth MTF) T - transverse diameter; V - vertical diameter

The parameters measured were: a) length, breadth, and height of cuboid bone; b) shape and diameters (transverse and vertical) of calcaneal articular facet (CAF), fourth metatarsal facet, fifth metatarsal facets, ecto-cuneiform articular facet (ECAF), navicular articular facet (NAF), and os peroneum facet; c) length, width, and depth of peroneal groove (Figure 1, 2).

The maximum distance between the anterior and posterior end was measured as cuboid length (Figure 1A); similarly, cuboid breadth was measured as the maximum distance between the medial and lateral surface (Figure 1A), and cuboid height was measured as the maximum distance between the superior and inferior surface (Figure 1B).

In addition, the distance between the cuneiform facet and the metatarsal facet on the medial side was measured (Figure 2). The above parameters were measured using a digital vernier caliper with a sensitivity of 0.1 mm. Each parameter was measured three times non-consecutively, and the average was taken as the final measurement and imported to Microsoft Excel (Microsoft, Redmond, Washington). Statistical analysis was done using SPSS version 20 (IBM Inc., Armonk, New York). All the quantitative parameters were expressed as the mean and standard deviation. Independent t-test was used to calculate the differences at a 95% confidence interval, and a p-value of 0.05 or less was considered significant.

## Results

The morphometric data from the 103 cuboid bones (right 50, left 53) were analyzed, and the statistical analysis of all the parameters is shown in Table 1, as is the shape of each articular facet in Table 2.

**Table 1:** Morphometry of right and left cuboid bones CAF - calcaneal articular facet; MTF - metatarsal facet, ECAF - ecto-cuneiform articular facet, NAF - navicular articular facet a p-value of <0.05 was considered as statistically significant and signified by *

Parameters		Right (n=50) mean ± SD (mm)	Left (n=53) mean ± SD (mm)	p-value	Total mean ± SD (mm)
Cuboid	Length	33.72 ± 2.57	33.65 ± 2.64	0.682	33.69 ± 2.61
	Breadth	25.24 ± 2.67	25.61 ± 3.07	0.738	25.43 ± 2.87
	Height	22.36 ± 2.15	23.69 ± 2.71	0.044*	23.03 ± 2.43
CAF	Transverse	23.58 ± 2.17	22.85 ± 2.62	0.229	23.22 ± 2.40
	Vertical	16.04 ± 1.52	15.9 ± 2.18	0.194	15.97 ± 1.85
ECAF	Transverse	15.11 ± 2.03	14.54 ± 2.11	0.57	14.83 ± 2.07
	Vertical	10.59 ± 1.79	10.43 ± 2.06	0.121	10.51 ± 1.93
NAF	Transverse	5.84 ± 2.32	5.34 ± 2.06	0.022*	6.78 ± 1.78
	Vertical	6.95 ± 1.83	6.50 ± 1.76	0.204	7.16 ± 2.08
Fourth MTF	Transverse	9.48 ± 1.63	9.65 ± 1.39	0.284	9.66 ± 1.51
	Vertical	14.66 ± 2.14	14.51 ± 2.33	0.218	14.59 ± 2.24
Fifth MTF	Transverse	12.65 ± 3.08	12.41 ± 1.78	0.238	12.53 ± 2.43
	Vertical	10.19 ± 1.49	10.25 ± 1.52	0.872	10.22 ± 1.51
Facet for os peroneum	Transverse	9.81 ± 2.1	10.24 ± 1.73	0.603	10.03 ± 1.92
	Vertical	7.22 ± 1.51	7.08 ± 1.41	0.587	7.15 ± 1.46
Peroneal groove	Length	20.18 ± 2.08	20.13 ± 2.46	1.176	20.16 ± 2.27
	Breadth	8.31 ± 1.5	9.01 ± 1.52	0.01*	8.66 ± 1.51
	Height	4.09 ± 1.05	4.51 ± 1.17	1.309	4.30 ± 1.11
Distance between ECAF and MTF		6.82 ± 1.71	6.98 ± 1.31	0.35	6.88 ± 1.54

**Table 2:** Shape of the cuboid articular facets. CAF - calcaneal articular facet; MTF - metatarsal facet; ECAF - ecto-cuneform articular facet; NAF - navicular articular facet

Articular facets of cuboid	Right (n=50)		Left (n=53)	
	Shapes	No. of specimens	Shapes	No. of specimens
CAF	Saddle	50	Saddle	53
ECAF	Wedge	24	Wedge	21
	Circular	1	Circular	3
	Triangular	1	Triangular	1
	Oval	23	Oval	28
	Quadrilateral	1	Quadrilateral	0
NAF	Oval	12	Oval	8
	Circular	4	Circular	6
	Irregular	1	Irregular	0
	Wedge	2	Wedge	1
Fourth MTF	Rectangular	48	Rectangular	50
	Square	2	Quadrangular	3
Fifth MTF	Triangular	50	Triangular	50
Facet for os peroneum	Oval	35	Oval	17
	Round	1	Round	1
	Irregular	12	Irregular	35
	Wedge	2	Wedge	0

Cuboid bone

The mean length, breadth, and height of the cuboid bone were 33.69 ± 2.61 mm, 25.43 ± 2.87 mm, and 23.03 ± 2.43 mm, respectively. The length was higher on the right side, whereas the breadth was higher on the left side, with no statistical significance. However, the height of the cuboid bone was higher on the left and was statistically significant (0.044) (Figures 1A, 1B).

Calcaneal articular facet

The CAF was saddle-type on both sides, and the shape was either rectangular or reniform with posteromedial projection (Figure 2G). The mean transverse and vertical diameters were 23.22 ± 2.4 mm and 15.97 ± 1.85 mm, respectively. Even though the parameters were higher on the right side, it was not statistically significant.

Ecto-cuneiform articular facet

The shapes of the ECAF on the right and left sides, respectively, were wedge (48% and 39.62%), circular (2% and 5.66%), triangular (2% and 1.89%), oval (46% and 52.83%), and quadrilateral (2% and 0%). The mean transverse and vertical diameters were 14.83 ± 2.07 mm and 10.51 ± 1.93 mm, respectively (Figure 2E). The transverse and vertical diameters were larger on the right side but not statistically significant. On the medial surface of the cuboid, the ECAF was present at a mean distance of 6.88±1.54 mm from the margin of the metatarsal facet. The difference between the sides was not significant (p-value 0.35) (Figure 2).

Navicular articular facet

**Figure 3:**
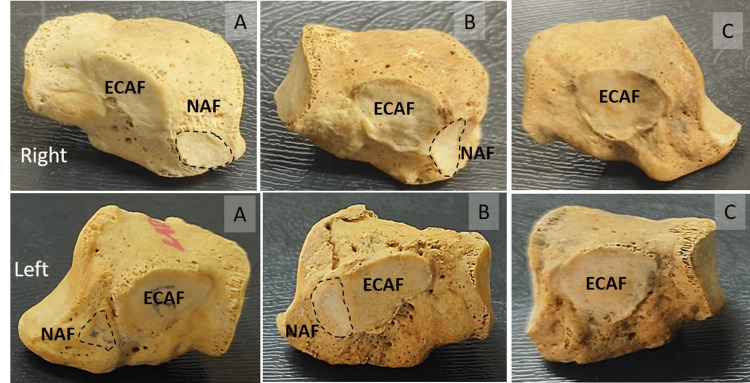
Medial view of navicular articular facet (NAF) of right and left cuboid bones A) separate facets for cuneiform (ECAF) and navicular bones; B) combined facet; C) navicular facet is absent

The shape of the NAF was oval (right 63.16%; left 53.3%), circular (right 21.05%; left 40%), irregular (right 5.26% and left 0%), and wedge (right 10.53; left 6.67%). NAF was present in 27 bones (26.2%). Separate navicular and cuneiform articular facets were observed in eight bones (right 5; left 3). Both the navicular and cuneiform bones had a combined facet with a ridge in-between in 19 bones (right 12; left 7) (Figure 3).

The range of the transverse diameter was 3.9 - 9.58 mm on the right side and 4.25 - 10.27 mm on the left. The range of the vertical diameter was 2.71 - 10.68 mm on the right and 2.78 - 8.33 mm on the left. The mean transverse and vertical diameters were 6.78 ± 1.78 and 7.16 ± 2.08 mm, respectively. The transverse diameter was higher on the right side and was statistically significant (p-value 0.02).

Fourth and fifth metatarsal articular facet

The shape of the fourth MTF was rectangular in 96%, and square shaped in 4% of the right-sided bones. On the left side, 94.34% were rectangular and 5.66% were quadrangular in shape. The mean transverse and vertical diameters were 9.66 ± 1.51 mm, and 14.59 ± 2.24 mm, respectively (Figure 2H). The transverse diameter was observed to be larger on the left side, and the vertical diameter was larger on the right side. However, they were not statistically significant.

The shape of the fifth MTF was triangular in 100% of the cuboid bones. The mean transverse and vertical diameters were 12.53 ± 2.43 mm and 10.22 ± 1.51 mm, respectively. Even though the transverse diameter was larger on the right side, and the vertical diameter on the left, they were not statistically significant.

Facet for os peroneum

Facet for os peroneum was observed in 74.76% (right 36.89%; left 37.86%). The shape of the facet was oval (right 70%; left 32.07%), round (right 2%; left 1.89%), irregular (right 24%; left 66.04%), and wedge (right 4%; left 0). The mean transverse and vertical diameters were 10.03 ± 1.92 mm, and 7.15 ± 1.46 mm, respectively (Figure 2F). In comparison between the sides, there was no significance noted in transverse and vertical diameters (p-value 0.603 and 0.587, respectively).

Peroneal groove

The mean length, breadth, and height of the peroneal groove were 20.16 ± 2.27 mm, 8.66 ± 1.51 mm, and 4.30 ± 1.11 mm, respectively (Figure 1C and 1D). The difference in breadth between the right and left sides was statistically significant (p-value 0.01), but the length and height were not (p-value 1.176 and 1.309, respectively).

## Discussion

The imbalanced kinetics and kinematics of the foot result in foot pain, which is a major complaint in the community. Exogenic and endogenic factors, including bone shape, morphometry, aging, sexual dimorphism, reduced physical activity, sunlight exposure, poor footwear, and obesity, could be the etiology. A comprehensive understanding of cuboid bone morphology is essential in the fields of anthropology, forensic medicine, and clinical medicine. This knowledge is essential in designing cuboid prostheses, performing foot reconstructive surgeries, correcting congenital deformities, and ensuring joint stability during lateral column lengthening osteotomies. Variations in the cuboid articular facets occur due to the evolutionary pressures on the cuboid bone and the molecular alterations at the time of development [8]. Thus, the dimensions of the cuboid articular facets will help to understand the biomechanics during gait and weight transmission in the body.

Length, breadth, and height of cuboid

The human foot is designed like a segmented lever made up of wedge-shaped bones and interconnecting ligaments, plantar fascia, and its modifications. This arrangement helps with grasping and gripping the foot while walking on uneven surfaces. During the stance phase of gait, the integration and congruency of the articular facets of the cuboid with the other tarsal bones maintain the lateral column of the foot in a closely packed position [9]. The cuboid bone determines the length of the lateral column of the foot, and the reduction of cuboid length in fractures results in the collapse of the lateral longitudinal arch. Knowledge regarding this is essential during the surgical management of cuboid fractures [10]. The summit of the lateral longitudinal arch lies low when the height of the cuboid is less, and this might be one of the causes of the flat foot [11]. Zhou et al. conducted flatfoot modeling using fresh-frozen cadavers and found that a 3 mm lateral column lengthening achieved a good correction for the flat foot through cuboid osteotomy [12]. Knowledge about the variations in the height of the cuboid can assist in evaluating plantar mid-foot ulceration and Charcot neuropathy [13, 14].

Harris et al. reported that the cuboidal breadth is one of the best parameters for sexual dimorphism [2]. The mean cuboidal breadth in their study was 26.29 ± 1.58 mm, whereas it was 25.42 ± 2.8 mm in the present study. The cuboidal length (33.69 ± 2.51 mm) and height (23.03 ± 2.43 mm) were comparatively higher in the present study than those of Viveka et al. (25.9 ± 2.45 mm and 22 ± 1.5 mm, respectively) [4]. A cuboidal height of 26.17 mm was comparatively higher in the study by Moitra et al. [15]. However, the height of the cuboid in the present study demonstrated a statistically significant difference between both sides.

Calcaneal articular facet

The calcaneo-cuboid (C-C) joint has the least mobility among the intrinsic foot joints, but it is highly variable between individuals [16]. The calcaneal process of the cuboid acts like a pivot at the C-C joint for medial and lateral rotation as a component of inversion and eversion [17]. In foot kinematics, the C-C joint transforms the midtarsal joint from a mobile grasping organ to a stable lever system during the walking cycle [18]. The morphometry of the C-C joint is important in planning surgeries involving mid-tarsal joint dislocation or fracture (calcaneo-cuboid or talonavicular joints) [19].

The most common pattern of the calcaneal articular facet was concavo-convex, which could be oval or reniform-shaped [15]. The mean transverse and vertical diameter of the calcaneal facet in the present study were 23.22 ± 2.39 mm and 15.97 ± 1.85 mm, respectively, and were comparable to the study by Moitra et al. (24.24 ± 2.19 and 16.45 ± 1.64 mm, respectively) [15].

Ecto-cuneiform and navicular articular facet

The longitudinal arches of the foot played a crucial role in the evolution of human bipedalism, shock-absorbing mechanisms, and weight transmission. Tazaki et al. suggested that cuboid osteotomy would be useful in lengthening the lateral column for flat foot correction. The osteotomy line on the cuboid should pass 4 mm from the calcaneocuboid joint laterally due to the attachment of the long plantar ligament, and on the medial side, 6 mm from the cuboid-metatarsal joint due to the presence of a navicular facet proximal to the cuneocuboid joint [7]. The presence or absence of a navicular facet depends on the type of gait pattern, weight transmission, and environmental and genetic factors. The mechanical load due to different gaits induces the formation of joint surfaces between the navicular and cuboid with variant morphology [20]. The angle between the navicular and cuboid articular facets is crucial for weight transmission and shock absorption during various physical activities such as jumping, jogging, etc. [20].

Viveka et al. observed separate articular facets for the navicular bone in 13 of the 22 bones examined [4]. In the present study, separate facets for navicular and cuneiform bones were observed in 8 of the 103 bones, and the morphometry of the navicular facet showed a significant difference between the right and left sides. Inefficient locking and aberrant articular configuration between the cuboid and navicular bones result in the weakness of peroneal muscle groups. The weakness of muscles and unstable articular surfaces affect the biomechanics of the foot and cause complications, including lateral column foot pain and osteoarthritis [21]. The cuboid-navicular coalition is rare and is associated with chronic foot pain syndrome. The morphology of the cuboid-navicular facet is useful in the direct diagnosis of the tarsal coalition as well as in correcting it [20]. Even though the navicular articular facet of the cuboid is inconstant, it plays an important role in biomechanics and various phases of the gait cycle. It also aids in the sex determination of unknown individuals using skeletal remains and helps in the biological profiling of an individual [22].

Fourth and fifth metatarsal facets

Among the tarsometatarsal joints, the mobility of the fourth and fifth metatarsals relative to the cuboid was greater than that of the first, second, and third metatarsals relative to the cuneiforms [16]. Gliding movement at the tarsometatarsal joint is necessary to walk on uneven surfaces and to support the lateral longitudinal arch during the transmission of body weight. Disruption in the soft tissue around the joint leads to instability, which results in chronic foot pain and gait abnormalities [23]. The dimensions of the joint surfaces for the fourth and fifth MT in the cuboid bone will aid in understanding the pathology and rectifying the lateral foot pain syndrome. In this study, the mean transverse and vertical diameters of the fourth and fifth MT facets were measured. These dimensions were 9.57 ± 1.51 mm and 12.53 ± 2.43 mm for the fourth MT facet, and 14.59 ± 2.24 mm and 10.22 ± 1.51 mm for the fifth MT facet, respectively. Moitra et al. studied the fourth and fifth MT facets together and found that the transverse diameter (13.85 mm) was less than the vertical diameter (21.32 mm). However, they did not address the fourth and fifth MT facets separately [15].

OS peroneum articular facet

The vestigial os peroneum occurs due to environmental stimuli and mechanical loads during daily activities. Guimera et al. have demonstrated that the os peroneum, either fibrous or fibrocartilaginous, develops during the embryonic period [24]. This supernumerary bony ossicle can induce lateral foot pain in addition to the os peroneum pathologies such as fracture, degenerative arthritis, nerve entrapment, and friction syndrome [24-26]. In this study, the mean transverse and vertical diameters of the os peroneum facet were 10.03 ± 1.92 mm and 7.40 ± 1.46 mm, respectively. The morphology and morphometry of the os peroneum facet are crucial while treating and reconstructing the peroneus longus sprain and rupture.

Peroneal groove

The arciform and frenular fibers in the sole convert the cuboid sulcus into the cuboid tunnel, the vulnerable site for the peronus longus tendon (PLT) injury due to high shear stress [27]. The position of PLT in the cuboid sulcus varies with different kinetics of the ankle and foot. Cho et al. reported that the dimensions of the cuboid tunnel determine the position of PLT in ultrasonography [28]. Similarly, Stone et al. observed that PLT was within the tunnel during plantar flexion and overlying it during dorsiflexion [29]. During its course, the PLT enters multiple tunnels; however, its position in the cuboidal tunnel is crucial due to its susceptibility to injury ranging from microtrauma to rupture [30].

In the present study, the mean length, breadth, and height of the peroneal groove were 20.16 ± 2.27 mm, 8.66 ± 1.51 mm, and 4.30 ± 1.11 mm, respectively. The mean depth of the peroneal groove was 0.636 mm in dried bone specimens in a study by Moitra et al. and 1.83 ± 1.3 mm by Viveka et al. [4, 15]. The variations in the dimension of the groove result in variable positions of the PLT, which could assist in understanding the PLT injury.

## Conclusions

Among all the tarsal bones, the cuboid is often neglected and lacks studies on its morphology. Though ignored, the contribution of the cuboid bone in foot kinematics is significant. The purpose of the study was to analyze the morphology of the cuboid bone in the South Indian population, and we hope that the morphometric data of the different facets of the cuboid bone might fill the lacunae in the literature. Although the study has limitations, such as unknown gender and age, it is aimed at providing additional information that may be useful in identifying unknown skeletal remains and designing tarsal bone prostheses for foot reconstruction. In addition, the morphometric data will aid in understanding congenital or acquired anomalies, pathological conditions, and mechanical dysfunctions.
